# Supplementary material for: Abacavir antiretroviral therapy and indices of subclinical vascular disease in persons with HIV
Source: PLoS One. 2022 Mar 10;17(3):e0264445. doi: 10.1371/journal.pone.0264445 (PMC8912137; doi:10.1371/journal.pone.0264445)
Supplement: Attachment — Submitted filename: ResponsetoReviewers_2.pdf [file pone.0264445.s002.pdf]

UHealth UNIVERSITY OF MIAMI HEALTH SYSTEM | UNIVERSITY OF MIAMI MILLER SCHOOL of MEDICINE

December 22nd,2021

Dear Dr. Huimin Yan,

Thank you for the review of our paper PONE-D-21-20487 “Abacavir Antiretroviral Therapy and Indices of Subclinical Vascular Disease in Persons with HIV”. My co-authors and I are appreciative of the quality feedback provided by the reviewers, which has helped to improve our manuscript. All modifications pertaining to the reviewer’s comments/suggestions are highlighted in the text of the paper in yellow. We have responded to each point raised by the reviewers. Below please find our a summary our responses, point-by-point.

Reviewer #1:
1. Could the authors explain why persons with didanosine or women postmenopausal were excluded.

Response*: an explanation has been inserted; see pg 3 para 1 lines 4-8*

2. A limitation of the study is that participants have not been matched by age a factor closely related with cardiovascular risk. I suggest to add this issue in the limitations.

Response*: comment has been added regarding this limitation; see pg 8 para 3 lines 8-12*

3. Could the authors define mildly and moderately increase of TG and TC. I suggest to use standard definitions: TG >150 mg/d Land TC>200 mg/dL.

Response: we have added these criteria in the footnotes of Table 1

Reviewer #2:
1. …the authors must present the estimated CVD risk on some scale, for example, ASCVD

Response: Thank you for raising this issue. As suggested the ASCVD 10-year risk score has been reported in the results, Table 1, and included in the discussion.

2. The conclusions of the abstract should indicate the characteristics of the population studied.

Response: as suggested; see Abstract line 19

3. It is not possible either to affirm with their results that ABC is not associated with increased CVD risk, which is a clinical variable that they have not analyzed. Instead, it is not associated with changes in FMD or rigidity or the number of EPC.

Response: as suggested ASCVD parameters have been inserted in results, table 1, and discussion.

4. Minor comment: slightly elevated triglycerides on table 1; from a clinical point of view, this may be easy to understand, but I suggest displaying the triglyceride values with their median and IQR.

Response: median and IQR values have been inserted for triglycerides and total cholesterol; see Table 1

Sincerely,

Claudia Martinez MD
